# Overexpression of Receptor for Advanced Glycation End Products and High-Mobility Group Box 1 in Human Dental Pulp Inflammation

**DOI:** 10.1155/2014/754069

**Published:** 2014-07-10

**Authors:** Salunya Tancharoen, Tassanee Tengrungsun, Theeralaksna Suddhasthira, Kiyoshi Kikuchi, Nuttavun Vechvongvan, Masayuki Tokuda, Ikuro Maruyama

**Affiliations:** ^1^Department of Pharmacology, Faculty of Dentistry, Mahidol University, 6 Yothe Road, Rajthevee, Bangkok 10400, Thailand; ^2^Department of Advanced General Dentistry, Faculty of Dentistry, Mahidol University, Bangkok 10400, Thailand; ^3^Department of Oral and Maxillofacial Surgery, Faculty of Dentistry, Mahidol University, Bangkok 10400, Thailand; ^4^Departments of Physiology and Neurosurgery, Kurume University School of Medicine, Fukuoka 830-0011, Japan; ^5^Department of Public Health and Environment, Nakhon Pathom Municipality, Nakhon Pathom 73000, Thailand; ^6^Department of Restorative Dentistry and Endodontology, Kagoshima University Graduate School of Medical and Dental Sciences, Kagoshima 890-8544, Japan; ^7^Department of Systems Biology in Thromboregulation, Kagoshima University Graduate School of Medical and Dental Sciences, Kagoshima 890-8520, Japan

## Abstract

High mobility group box 1 (HMGB1), a nonhistone DNA-binding protein, is released into the extracellular space and promotes inflammation. HMGB1 binds to related cell signaling transduction receptors, including receptor for advanced glycation end products (RAGE), which actively participate in vascular and inflammatory diseases. The aim of this study was to examine whether RAGE and HMGB1 are involved in the pathogenesis of pulpitis and investigate the effect of Prevotella intermedia (*P. intermedia*) lipopolysaccharide (LPS) on RAGE and HMGB1 expression in odontoblast-like cells (OLC-1). RAGE and HMGB1 expression levels in clinically inflamed dental pulp were higher than those in healthy dental pulp. Upregulated expression of RAGE was observed in odontoblasts, stromal pulp fibroblasts-like cells, and endothelial-like cell lining human pulpitis tissue. Strong cytoplasmic HMGB1 immunoreactivity was noted in odontoblasts, whereas nuclear HMGB1 immunoreactivity was seen in stromal pulp fibroblasts-like cells in human pulpitis tissue. LPS stimulated OLC-1 cells produced HMGB1 in a dose-dependent manner through RAGE. HMGB1 translocation towards the cytoplasm and secretion from OLC-1 in response to LPS was inhibited by TPCA-1, an inhibitor of NF-*κ*B activation. These findings suggest that RAGE and HMGB1 play an important role in the pulpal immune response to oral bacterial infection.

## 1. Introduction

Dental caries and subsequent tooth pulp inflammation (pulpitis) are major oral health issues caused by oral bacterial infection. Pulpitis accompanies the host's innate and adaptive immune responses to these bacteria [1]. Apart from a rich neurovascular supply, the pulp cavity is home to odontoblasts (the cells that form dentin in the tooth) which are involved in innate immunity against dentin-invading pathogens and are the first to encounter the caries bacterial antigens. Odontoblasts are also suspected of being involved in tooth development and mineralization [2], maintenance of the pulp immune, and inflammatory responses to dentin-invading pathogens [3] as well as undergoing apoptosis [4].

Anaerobic gram-negative bacteria, including* Prevotella intermedia* (*P. intermedia*), have been implicated in human dental pulp inflammation and periapical diseases, which are associated with rapid pulp degeneration, necrosis, and destruction of periapical tissue.* P. intermedia* has been shown previously to play a key role in periapical tooth disease and in the maxillofacial abscess formation via the expression of inflammatory cytokines [5]. LPS has the ability to trigger a number of host cells, especially mononuclear phagocytes, to produce and release a wide variety of pharmacologically active mediators, including tumor necrosis factor (TNF)-*α*, interleukin (IL)-1*β*, IL-6, and IL-8 [6]. In addition, it can induce dental pulp fibroblasts to release IL-6 [7] and IL-8 [8]* in vitro*. These cytokines have previously been implicated in the pathogenesis of pulpitis [9–11]. While monocytes or fibroblasts in dental pulp tissues have been found to express proinflammatory cytokines, it is not clear whether odontoblasts are involved in the host response and/or produce inflammatory mediators upon stimulation with LPS from* P. intermedia*.

Receptor for advanced glycation end products (RAGE) is a member of the immunoglobulin superfamily and is expressed on mononuclear phagocytes, vascular smooth muscle cells, and neurons [12, 13]. RAGE interacts with a range of ligands, including advanced glycation end products (AGEs), high-mobility group box 1 (HMGB1), and S100/calgranulins [14–16]. Ligand binding results in RAGE-dependent sustained NF-*κ*B activation, which perpetuates the inflammatory response [17]. RAGE is expressed at low levels in normal tissues and in the vasculature and is upregulated in the diabetic vasculature or at other sites where its ligands accumulate [18].

HMGB-1 is a ubiquitous 25 kDa nuclear DNA-binding protein that, under normal conditions, is located in the cell nucleus, where it organizes the chromatin structure, DNA replication, and transcription. However, HMGB1 also acts in the extracellular environment as a primary proinflammatory signal. Upon HMGB1 stimulation, human microvascular endothelial cells respond by upregulating adhesion molecules and producing of proinflammatory cytokines [19]. Thus HMGB1 is not only released in response to proinflammatory stimuli, but also induces the production of inflammatory mediators [20] and expression of adhesion molecules [19]. This suggests that HMGB1 can propagate an inflammatory response during infection or injury. In addition, HMGB1 is known to contribute to the pathogenesis of various inflammatory diseases [21–23], including periodontal disease [24]. Furthermore, the blockade of HMGB1 release using an anti-HMGB1 monoclonal antibody, competitive antagonist, or short hairpin RNA has already been shown to be effective in various animal models of disease including traumatic brain injury [25], stroke [26], rheumatoid arthritis [27], acute pancreatitis [28], and cancer [29].

Although RAGE and HMGB1 have been located in multiple inflamed human tissues, their presence in inflamed dental pulp tissue has not been elucidated. To the best of our knowledge, there have been no reports of how RAGE and HMGB1 contribute to the pathogenesis of human pulpitis. Thus, the purpose of this study was to investigate the expression of RAGE and HMGB1 in human dental pulpitis and to examine RAGE and HMGB1 production, mediated through NF-*κ*B activation, in odontoblast-like cells stimulated with LPS from* P. intermedia*.

## 2. Materials and Methods

### 2.1. Reagents

HMGB1 antibody (Ab) was obtained from SHINO-TEST (Tokyo, Japan). RAGE Ab was purchased from Abcam (Cambridge, MA, USA). A potent inhibitor of IKK-*β* (IKK-2), TCPA-1 (IKK-2 inhibitor IV), was purchased from Santa Cruz Biotechnology (Santa Cruz, CA, USA). Unless otherwise stated, all other reagents were supplied by Sigma-Aldrich Inc. (St. Louis, MO, USA).

### 2.2. Preparation of LPS from Prevotella Intermedia (*P. intermedia*)

*P. intermedia* (ATCC 25611) was cultured in GAM broth (Nissui Seiyaku Co., Tokyo, Japan) at 37°C for 18 h in a N_2_ : H_2_ : CO_2_ (85 : 10 : 5) atmosphere in an anaerobic culture system (MIP-1025; Sanyo, Tokyo, Japan). LPS was then prepared by the hot phenol-water extraction method [8, 30]. The chemical analysis of the LPS obtained was similar to that reported previously by Hamada et al. [31].

### 2.3. Preparation of Human Dental Pulp Tissues

This study was conducted with the approval of the Committee on Human Rights Related to Human Experimentation, Mahidol University. Participants were informed of risks and benefits and signed an approved informed consent document prior to enrollment. Written informed consent was obtained from each patient. Dental pulp tissues were obtained from 22 patients of both sexes, who did not take antibiotics for 3 weeks previously and did not have systemic diseases, no periapical lesions, and no loss of periodontal attachment. Healthy human dental pulp samples were collected from teeth extracted for orthodontic reasons or from third molars having a clinical diagnosis of nonoccluded teeth (*n* = 15). Human dental pulpitis tissues were collected from teeth having a clinical diagnosis of irreversible pulpitis (*n* = 15). These patients were suffering from spontaneous pain of approximately 24 h duration. Extracted teeth were immediately submerged in the RNA stabilizing solution, RNA Later (Sigma, UK). Teeth were subsequently longitudinally sliced, using a segmented, diamond-edged rotary saw (TAAB Laboratories, Berkshire, UK) and cooled with PBS, and the pulpal tissue was carefully removed intact using a sterile dental probe and forceps. This technique has previously been shown to provide core pulpal tissue with the odontoblast layer left intact on the dentin surface [32, 33].

### 2.4. Odontoblast-Like Cell Culture and LPS Stimulation

Odontoblast-like cells, OLC-1, obtained from mouse tooth germs were provided by Dr. Toshihiro Sugiyama (Department of Biochemistry, School of Medicine, Akita University, Akita, Japan) and were maintained in minimum essential medium alpha modification (*α*-MEM) (Sigma, USA) supplemented with 15% (v/v) heat-inactivated fetal bovine serum (Sigma. USA) and fibroblast growth factor (FGF)-2 (2 ng/mL) and were grown on type-I collagen coated culture plates at 37°C in a humidified chamber with 5% (v/v) CO_2_ in air as described previously by Arany et al. [34].

For RNA and protein extractions, cells were cultured in 60 mm dishes (1 × 10^6^ cells/mL) and serum-starved with serum-free Opti-MEM-I medium (Gibco, Grand Island, NY, USA) for 24 hours. Cells were then stimulated with LPS as indicated in a time-and dose-dependent assay. The supernatant was collected and stored at −80°C until use and then, after washing with sterile PBS, the cells were harvested for either RNA or for protein extraction.

### 2.5. Silencing of RAGE Gene Expression and Reverse Transcriptase-Polymerase Chain Reaction (RT-PCR) Detection for RAGE mRNA and HMGB1 Release

RNA silencing was performed with small interfering RNA (siRNA) targeting mouse RAGE mRNA or negative control siRNA (Santa Cruz, catalog: sc-36375). Cells were transfected with siRNA duplexes suspended in lipofectamine reagent (Life Technologies) following the manufacturer's protocol. Briefly, cells were cultured in 6-well plates until 60% confluence. Cells were washed with serum-free Opti-MEM I Reduced Serum Medium (Gibco, Grand Island, NY). RAGE siRNA and negative control siRNA were gently premixed with lipofectamine reagent in Opti-MEM medium for 20 min at RT. The siRNA (final concentration 100–200 nM)/lipofectamine reagent complex was overlaid onto the washed cells for an additional 48 hours at 37°C in 5% CO_2_. The efficacy of gene silencing was evaluated using RT-PCR. For extracellular HMGB1 release measurement, cells were transfected with siRNA as indicated and stimulated with LPS for 24 h, and the supernatant was collected and stored at −80°C until use.

Total RNA was extracted using TRIzol-Reagent according to the manufacturer's instruction (Invitrogen, Carlsbad, CA). First-strand cDNA was synthesized by reverse transcriptase using a commercial kit (Takara Biomedicals, Tokyo, Japan), and the reaction was performed following the manufacturer's instructions. The resulting cDNA mixture was amplified with Taq polymerase. The primer sequence for mouse RAGE mRNA detection was 5′-CCTGGGTGCTGGTTCTTGCTCT-3′ and 5′-GATCTGGGTGCTCTTACGGTCC-3′ (nucleotides 31–52 and 1209–1230 in GenBank L33412) and GAPDH mRNA was 5′-GTCTTCCTGGGCAAGCAGTA-3′ and 5′-CTGGACAGAAACCCCACTTC-3′. The numbers for amplification cycles were 30 cycles. The amplification products were electrophoresed through 2% agarose gel containing ethidium bromide. Cell viability was monitored after incubation for 24, 36, and 48 hours by 3-[4,5]-2,5-diphenyltetrazolium bromide (MTT) assay. Briefly, cells were incubated with MTT (0.5 mg/mL; final concentration) for 3 h. Formazan product was solubilized by the addition of dimethyl sulfoxide for 16 h. Dehydrogenase activity was expressed as absorbance at a test wavelength of 570 nm and at a reference wavelength of 630 nm.

### 2.6. Quantitative Real-Time PCR Analysis

The expression of selected genes in dental pulp tissues and LPS-stimulated OLC-1 cell cultures was measured by quantitative real-time reverse transcription (RT) PCR as previously described [23]. Total RNA was extracted from a pool of three dental pulp tissues from pulpitis or healthy samples using TRIzol Reagent (Invitrogen, Carlsbad, CA, USA). Samples (2 *μ*g) of total RNA were reverse transcribed using a First Strand complementary DNA synthesis kit for RT-PCR (Roche, Indianapolis, IN, USA). cDNA was amplified by real-time RT-PCR (Ct value 20–30 s cycles) using a 7300 Real-Time PCR System (Applied Biosystems, Foster City, CA, USA) with gene-specific primers (assay IDs: receptor for advanced glycation end products (RAGE), Hs00542584_g1 for human and Mm00545815 m1 for mouse; glyceraldehyde-3-phosphate dehydrogenase [GAPDH], Hs99999905_m1 for human and Mm99999915-g1 for mouse). As “minus RT” controls, samples containing total RNA instead of the cDNA were examined. The TaqMan technique was used for signal detection [35]. All analyses were carried out in triplicate, and nontemplate controls and dissociation curves were used to ensure the specificity of template amplification. For each primer pair, serial dilutions of a control cDNA were used to construct standard curves, and those with *R* ^2^ > 0.97 were then used to determine mRNA levels in individual samples. The mRNA levels for each gene of interest were normalized to the GAPDH mRNA levels in the same cDNA sample. For OLC-1 cell culture, the real-time RT-PCR assays were repeated as above, with the exception of a different RAGE primer (assay ID, Mm00545815_m1) [36].

### 2.7. Immunohistochemistry

After tissues were fixed with formalin buffer, paraffin-embedded sections were deparaffinized in xylene and rehydrated through a series of decreasing concentrations of ethanol. Staining was carried out using the indirect immunoperoxidase diaminobenzidine (DAB) method. Endogenous peroxidase was blocked by 0.3% H_2_O_2_ for 5 min. Sections were incubated for 1 h at room temperature (RT) with polyclonal anti-RAGE Ab (2 *μ*g/mL) in Ab diluent with background reducing components (DakoCytomation, Carpinteria, CA, USA). As a negative control, isotype-matched control IgG was used at the same concentration. After rinsing with PBS, sections were finally developed with a DAKO LSAB+ System, HRP (DakoCytomation; KO679), and immunostaining was visualized with substrate solution (DAB). Counterstaining was performed with Mayer's hematoxylin.

### 2.8. Immunofluorescence

Immunofluorescence analyses were carried out as described previously [37]. After tissues were embedded in paraffin, 6-micron thick sections were cut, deparaffinized, and rehydrated. The slides were blocked with 1% BSA in PBS containing 0.1% Triton X-100. After washing, the slides were incubated with anti-HMGB1 rabbit polyclonal Ab at room temperature for 1 h. After further washing, the slides were incubated with Alexa Fluor 488-labeled goat anti-rabbit IgG (1 : 200), washed again, and stained with DAPI. Cells were visualized under an Axioskop fluorescence microscope (Carl Zeiss, Oberkochen, Germany).

### 2.9. Preparation of Tissue and Cells for Western Blot Analysis

#### 2.9.1. Preparation of Tissue and Whole-Cell Lysates

Tissues and whole-cell lysates were prepared for RAGE protein expression as per the standard protocol. Briefly, tissues were homogenized with a Polytron homogenizer using ice-cold lysis RIPA buffer (1% Nonidet P-40, 0.5% sodium deoxycholate, 0.1% SDS in PBS) containing protease inhibitors (Complete, Roche). Homogenates were centrifuged at 10,000 g for 10 min at 4°C, and supernatants were collected for immunoblots. For the preparation of whole-cell lysates, cells were washed with PBS and centrifuged (2000 g × 10 min). The resulting cell pellet was subsequently processed for immunoblots.

#### 2.9.2. Preparation of Nuclear and Cytosolic Fractions

HMGB1 protein expression in the nucleus and cytoplasm was determinedusing a compartmental protein extraction kit (Chemicon) following the manufacturer's instruction. Tissues were weighed and homogenized with buffer C at moderate speed for 20 sec. Samples were then stood on ice for a few seconds and homogenization was repeated two more times. The mixture was then rotated at 4°C for 20 min. Then, the samples were centrifuged at 18,000 g at 4°C for 20 min. The cytoplasmic proteins are contained in the supernatant. The pellets were then resuspended in buffer W and spun a second time at 18,000 g for 20 minutes at 4°C. The pellets were placed in buffer N, rotated, and spun again as described above. Aliquots of the supernatant containing nuclear and cytosol proteins were kept for further investigation.

#### 2.9.3. Western Blot Analysis

Protein concentrations were determined by Bradford protein assay using bovine serum albumin as standard (Bio-Rad, Hercules, CA, USA). Samples were mixed with 2× electrophoresis sample buffer solution with bromophenol blue (Santa Cruz Biotechnology) before being subjected to 12% SDS-polyacrylamide gel electrophoresis (PAGE) and transferred onto nitrocellulose membranes (Schleicher & Schuell, Dassel, Germany). Samples containing 10 or 15 *μ*g of total protein were used. To prevent nonspecific binding, the membrane was blocked with a solution containing 5% (w/v) nonfat dry milk with 1% (v/v) Tween 20 in PBS for 1 hour at RT. Rabbit anti-HMGB-1 or RAGE primary antibodies were incubated for 3 h at RT and overnight at 4°C, respectively. Then the membranes were washed and incubated with horseradish peroxidase-conjugated anti-rabbit polyclonal IgG (MP Biomedicals Inc., Solon, OH, USA) at RT for 1 h. Labeled bands were visualized using an enhanced chemiluminescence system (GE Healthcare Bio-Science, Pittsburgh, PA, USA) and exposed to high-performance chemiluminescence film (GE Healthcare). The intensity of the protein bands in western blotting was quantified using National Institutes of Health Image 1.63 software.

### 2.10. Flow Cytometric Analysis

OLC-1 monolayers were gently dispersed and resuspended at a final concentration of 3 × 10^6^ cells/mL and FACS was performed as described previously [38] with slight modifications. After washing with PBS, cells were fixed with OptilyseC (Becton Dickinson, Franklin Lakes, NJ, USA). Next, cells were washed with PBS and incubated with the RAGE Ab or isotype-matched control (2 *μ*g/mL), at 4°C for 1 h, followed by the fluorescein isothiocyanate (FITC-) conjugated secondary Ab (ICN Pharmaceuticals, Aurora, OH, USA) for 30 min in the dark. Fluorescence was analyzed with a FACScan analyzer (Beckman Coulter, Fullerton, CA, USA).

### 2.11. Proinflammatory Cytokines Measurement by Enzyme-Linked Immunosorbent Assay (ELISA)

HMGB1 levels in the cytosol fraction of the tissue and cell culture supernatant were quantified using a commercial kit (Shino-test, Sagamihara, Kanagawa, Japan). The presence of interleukin (IL)-1*β* and IL-8 in the cell culture supernatant was determined by ELISA using a commercial kit (BioSource, Camarillo, CA, USA).

### 2.12. Statistical Analysis

Experimental values are given as the mean ± S.D. Statistical significances between different groups were assessed by one-way analysis of variance (ANOVA) test or Student's paired *t*-test using Sigma Stat for Windows, version 3.5 (Systat Software, Inc., Chicago, IL, USA). *P* values <0.05 were considered statistically significant.

## 3. Results

### 3.1. Overexpression of RAGE in Human Pulpitis Tissue

**Figure 1:**
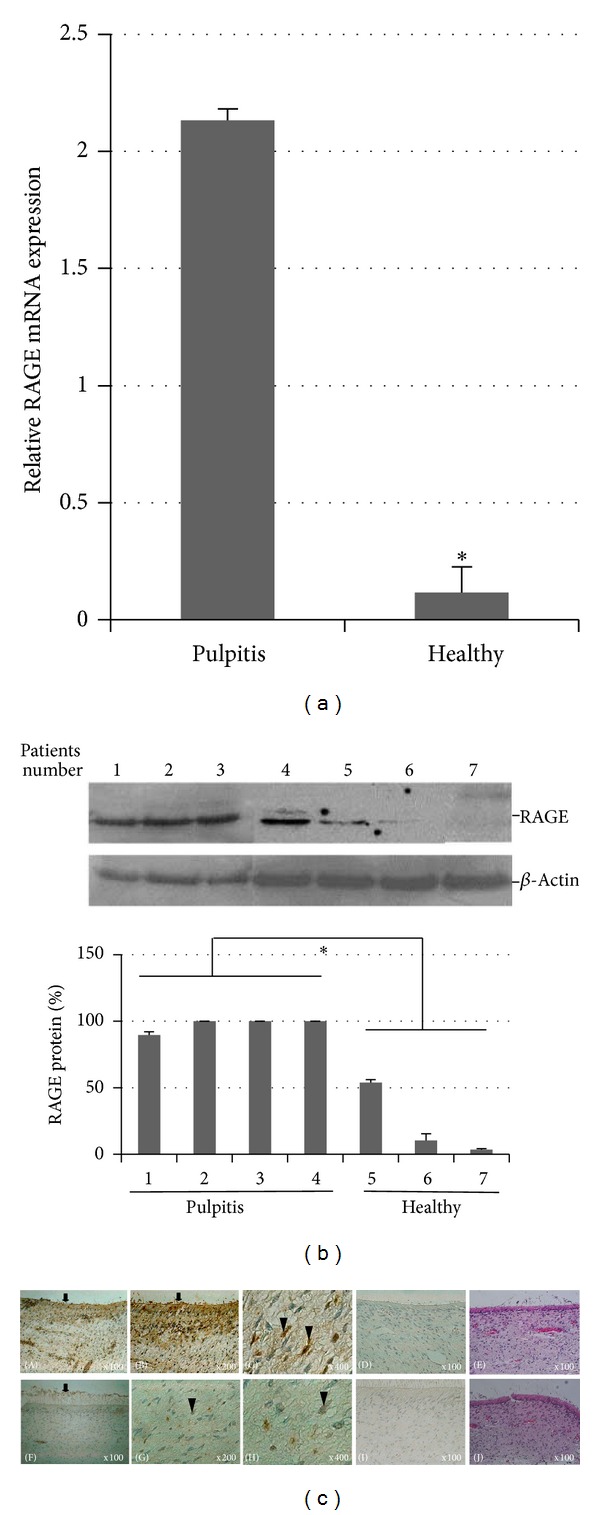
Upregulated expression of RAGE in human pulp tissue from teeth with a clinical diagnosis of irreversible pulpitis. (a) The expression of RAGE in inflamed human and healthy tissue was examined by quantitative real-time PCR. Total RNA was extracted from a pool of three dental pulp tissues from either pulpitis or healthy samples. RAGE mRNA levels were quantified relative to levels of GAPDH. (b) RAGE protein in inflamed and healthy pulp tissues was examined by western blot analysis. Representative blots using antibodies against RAGE and *β*-actin from four pulpitis and three healthy pulp tissues are shown. RAGE expression was quantified by densitometry and normalized to *β*-actin. Results are expressed as the means ± SD from three independent experiments. *P < 0.001. (c) Immunochemical staining of RAGE in pulp tissue sections. Black arrows indicate odontoblast processes in tubules of predentin. Higher magnification of stromal pulp fibroblasts-like cells is indicated by arrowhead. RAGE expression in infiltrating inflammatory cells (∗). The IgG isotype control was employed at the same time and concentration (D and I) as the test antibody. Counterstaining was performed with Mayer's hematoxylin (E and J).

The expression of RAGE in inflamed human pulp tissue from teeth with a clinical diagnosis of irreversible pulpitis and healthy tissue was examined by quantitative real-time PCR, western blot analysis, and immunochemical staining. The relative RAGE mRNA expression was 2.13 ± 0.048 (2.09–2.18) versus 0.12 ± 0.10 (0.04–0.24) in pulpitis versus healthy tissues, respectively (*P* < 0.001, Figure 1(a)). Western blot analysis of 4 samples pooled from 15 different pulpitis patients or 3 samples pooled from 15 healthy tissues were probed using a specific anti-RAGE Ab (Figure 1(b)). Extracted proteins from both tissues resulted in the detection of a band of approximately 47 kDa. A strong RAGE signal was found in the pulpitis tissues (lanes 1–4), whereas weak protein signal was seen in the healthy tissue extracts (lanes 5–7). RAGE protein quantity, normalized to *β*-actin, showed significant differences in RAGE expression in pulpitis compared with healthy tissues (*P* < 0.001).

To confirm RAGE localization in these tissues, we stained tissue sections with the same RAGE Ab as was used for western blotting. Immunohistochemistry studies revealed RAGE expression was present in both healthy and pulpitis tissue.  Figure 1(c) showed abundant RAGE labeling observed in both odontoblastic and subodontoblastic cell layers (A and B), as well as in odontoblast processes in tubules of the predentin (A and B, arrow), stromal pulp fibroblasts-like cells (C, arrowhead), and endothelial-like cell lining in the pulpitis tissues (A and B). RAGE expression in infiltrating inflammatory cells in the interstitial connective tissues was intensely stained (∗). In healthy pulp tissues, RAGE signal was low in both the odontoblastic and the subodontoblastic cell layers (F, arrow), stromal pulp fibroblasts-like cells (G and H, arrowhead), and endothelial-like cell lining (F). No immunoreactive cells were stained with the IgG isotype control (D and I). These results led us to consider that increased RAGE expression might have a role in the regulation of pathologies of tooth pulp disease.

### 3.2. Abundant HMGB1 Expression in the Cytoplasmic Extracts of Human Pulpitis Tissues

**Figure 2:**
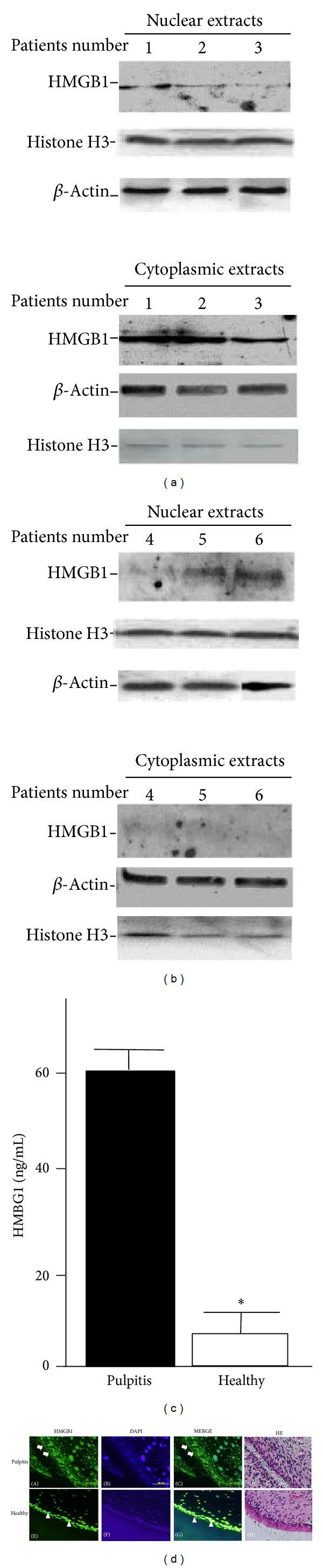
HMGB1 protein expression in the cytoplasmic extracts of human pulpitis tissues. Nuclear and cytoplasmic extracts from (a) pulpitis and (b) healthy tissues were determined by western blot analysis (*n* = 3). Histone and *β*-actin were used as loading controls for nuclear and cytoplasmic extracts. Note that HMGB1 translocates to the cytoplasmic fraction in the inflamed pulp tissues. (c) HMGB1 levels in the cytosolic fraction from both tissues were determined by ELISA. Results are expressed as the means ± SD from duplicate experiments. *P < 0.001. (d) Immunofluorescence microscopy showing the localization of cytoplasmic HMGB1 in the odontoblasts (white arrow) of pulpitis tissues. HMGB1 immunoreactivity was also seen in stromal pulp fibroblasts-like cells and endothelial-like cell lining (∗) in pulpitis tissues. HMGB1 was located in the nuclei of the odontoblasts (arrowhead) in healthy tissues. Blue depicts the nucleus stained with DAPI and green depicts the localization of HMGB1. Counterstaining was performed in Mayer's hematoxylin (D and H). Scale bar: 100 *μ*m.

The expression of HMGB1 in the nuclear and cytoplasmic extracts of pulp tissue samples from 3 separate patients with pulpitis (Figure 2(a), patients 1–3) and 3 healthy control subjects (Figure 2(b), patients 4–6) was determined by western blot analysis. Our findings demonstrated a distinct translocation of nuclear HMGB1 (upper panel) to the cytoplasm (lower panel) in tissues from the pulpitis patients (Figure 2(a)), whereas HMGB1 was present only in the nuclei of healthy tissues (Figure 2(b)). Accordingly, the cytosolic HMGB1 level in inflamed pulp tissues was significantly higher than that in healthy tissues (*P* < 0.001) as measured by ELISA (Figure 2(c)). To confirm the findings of HMGB1 translocalization described above, we examined whether increased expression of HMGB1 in the cytosol was visible in pulpitis tissues* in situ*. In immunofluorescence studies, pulpitis tissues revealed capillaries forming a coarse vascular network under the continuous layer of odontoblasts (Figure 2(d)). Strong cytoplasmic HMGB1 signal was noted in both odontoblastic and subodontoblastic cell layers (A and C, arrow), as well as in odontoblast processes in tubules of the predentin. HMGB1 signal was seen in the stromal pulp fibroblast-like and endothelial-like cell lining (A, C and D, ∗). HMGB1 was localized to the nuclei of both odontoblastic and subodontoblastic cell layers in healthy tissue (E and G, arrowhead). No HMGB1 signal was seen in odontoblast processes (E and G). Simultaneous incubation with anti-rabbit IgG had no cumulative inhibitory effect (data not shown). Our results demonstrated that HMGB1 translocated from the nuclei to the cytoplasm and was then secreted out from pulpitis tissue.

### 3.3. Effect of *P. intermedia* LPS on RAGE Production and Proinflammatory Cytokines Release in Odontoblast-Like Cells

**Figure 3:**
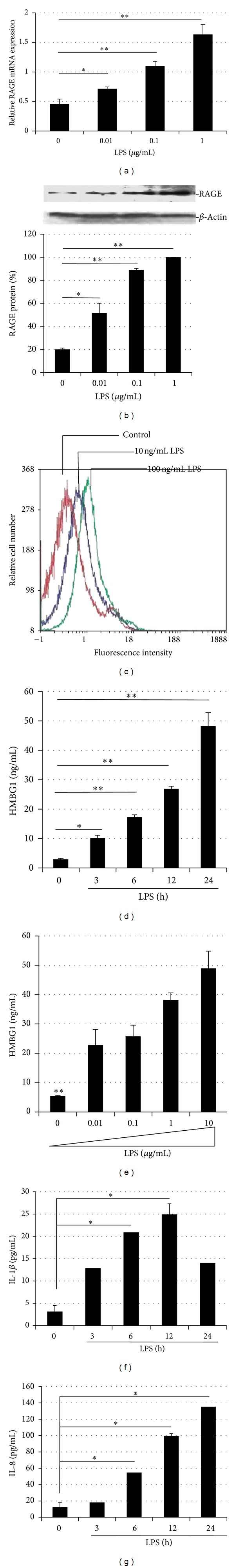
LPS upregulated RAGE expression and proinflammatory cytokines release in OLC-1 culture. (a) Cells were stimulated with LPS as indicated for 6 h. The expression of RAGE was examined by quantitative real-time PCR. (b) Cells were stimulated with LPS for 24 h, and RAGE protein was examined by western blot analysis. A representative blot using antibodies against RAGE and *β*-actin is shown. RAGE expression was quantified by densitometry and normalized to *β*-actin. (c) Increased RAGE surface expression by OLC-1 cells following LPS stimulation for 12 h was analyzed by flow cytometry. A representative histogram is shown. (d) Active release of HMGB1 in a time-dependent manner upon stimulation with 100 ng/mL LPS. (e) HMGB1 release in a dose-dependent manner after LPS stimulation for 24 h. IL-1*β* (f) and IL-8 (g) release in a time-dependent fashion following LPS stimulation. Results are expressed as the means ± SD from triplicate experiments. *P < 0.05, **P < 0.001.

The odontoblast-like cell line OLC-1 was incubated with various concentrations of LPS. We observed that RAGE mRNA production increased in OLC-1 following LPS treatment (Figure 3(a)). RAGE expression was first seen when LPS was added at 0.01 *μ*g/mL (*P* = 0.015 versus untreated cells) and gradually increased at higher doses (*P* < 0.001 versus untreated cells). Increased RAGE expression was also observed by western blot analysis and flow cytometry (FACS) analysis. As shown in Figure 3(b), LPS significantly enhanced RAGE protein production in a dose-dependent fashion compared with the untreated cells (*P* < 0.05). Accordingly, FACs analysis also demonstrated that addition of LPS induced an increase of RAGE protein expression (Figure 3(c)). The addition of LPS for 3–24 h also induced an increase in HMGB1 released into the cell culture supernatant in a time-dependent fashion as determined by ELISA (Figure 3(d)). LPS (100 ng/mL) stimulation for 3 h significantly induced HMGB1 release (10.15 ± 0.94 ng/mL) compared with the untreated cells (*P* = 0.002) and gradually increased following 24 h stimulation to 48.24 ± 4.57 ng/mL (*P* < 0.001 versus untreated cells). Furthermore, LPS stimulation for 12 h induced HMGB1 release into the cell culture supernatant in a dose-dependent manner (Figure 3(e)). The addition of 0.01 *μ*g/mL LPS significantly induced HMGB1 release (22.8 ± 5.3 ng/mL) which was increased gradually to 48.94 ± 5.8 ng/mL upon 10 *μ*g/mL LPS stimulation (*P* < 0.001 versus untreated cells). In addition, LPS induced IL-1*β* (Figure 3(f)) and IL-8 (Figure 3(g)) release from OLC-1 in a time-dependent manner. LPS significantly induced IL-1*β* (24.9 ± 9.6 pg/mL) and IL-8 (135.3 ± 23.3 pg/mL) release upon 12 h and 24 h stimulation, respectively, (*P* < 0.05 versus untreated cells).

### 3.4. Reduction of HMGB1 Release following *P. intermedia* LPS Stimulation in RAGE Knockdown OLC-1

**Figure 4:**
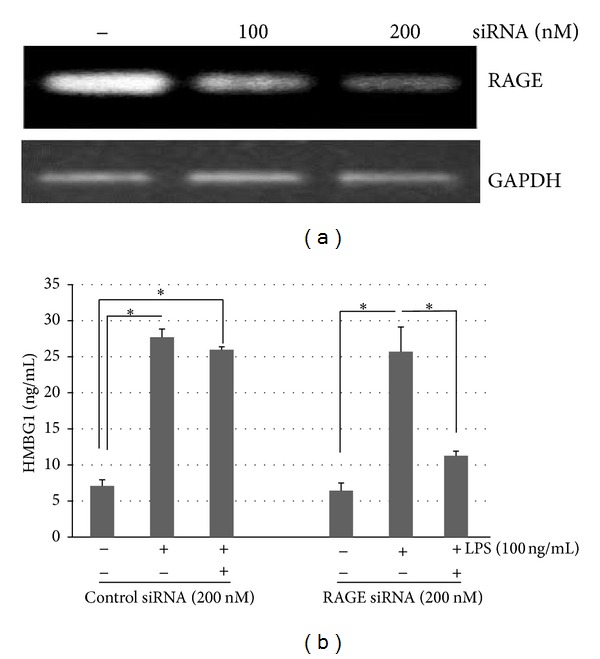
RAGE mediates the effects of LPS-induced HMGB1 release in OLC-1. Cells were transfected with RAGE siRNA suspended in lipofectamine reagent for 48 h. (a) By RT-PCR, RAGE siRNA silences RAGE mRNA expression without affecting house-keeping gene (GAPDH) mRNA expression. A representative experiment is shown, and the RAGE-specific RNA bands are indicated. (b) Cells were transfected with either RAGE siRNA or negative control siRNA and stimulated with LPS for 24 h, and the HMGB1 release in the supernatant was measured by ELISA. Results are expressed as the means ± SD from duplicate experiments. *P < 0.05.

The silencing of RAGE gene expression was carried out following the transfection of RAGE-specific siRNA in the presence of LPS in OLC-1 (Figure 4(a)). RT-PCR showed that RAGE siRNA markedly decreased the expression of RAGE mRNA without affecting house-keeping gene (GAPDH) expression or any toxicities after transfection (MTT assay; data not shown). Transfection of OLC-1 with 200 nM siRNA against RAGE suppressed LPS-induced HMGB1 release in the supernatant by ~57% compared with control siRNA (Figure 4(b)). These results thus suggested that knockdown of RAGE prevents the effect of LPS-induced HMGB1 release in OLC-1.

### 3.5. LPS Mediated HMGB1 Expression Involves NF-*κ*B Activation in OLC-1 Cells

**Figure 5:**
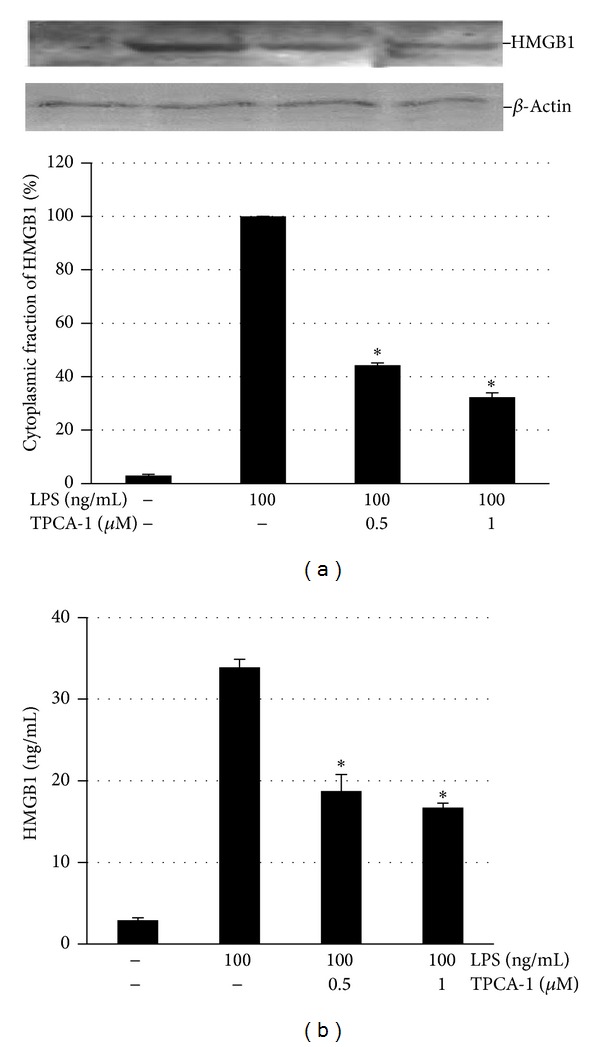
Inhibition of NF-*κ*B activation attenuated LPS-induced HMGB1 translocation from the nucleus to the cytoplasm and release in OLC-1. Cells were pretreated with the selective inhibitor of human IKK-2 (TPCA-1), 2 h before the cells were stimulated with LPS for 24 h. (a) The cells were harvested, and the cytoplasmic proteins were isolated for analysis of HMGB1 by western blotting. A representative blot using antibodies against HMGB1 and *β*-actin is shown. HMGB1 expression was quantified by densitometry and normalized to *β*-actin. (b) HMGB1 release into the supernatant was analyzed by ELISA. Results are expressed as the means ± SD from triplicate experiments. *P < 0.001.

To evaluate the signal transduction pathways involved in LPS-mediated HMGB1 expression, cells were pretreated with 0.5 and 1 *μ*M TPCA-1, a potent inhibitor of IKK-*β* (IKK-2) [39], and LPS-induced HMGB1 expression was evaluated by western blot analysis and ELISA. Preincubation with 0.5 and 1 *μ*M TPCA-1 inhibited LPS-induced cytoplasmic translocation of HMGB1 by 55% and 65%, respectively, (Figure 5(a)). Accordingly, TPCA-1 inhibited LPS-induced HMGB1 release into the supernatant was shown by ELISA (Figure 5(b)). TPCA-1 treatment alone had no effect on HMGB1 expression and release (data not shown).

## 4. Discussion

The present study has shown that RAGE and cytoplasmic HMGB1 are upregulated in pulpitis tissue. Furthermore, LPS from* P. intermedia* induced RAGE expression in odontoblast-like (OLC)-1 cells and the cytoplasmic translocation of HMGB1 prior to release into the supernatant, which was mediated through RAGE and NF-*κ*B activation.

Inflammation is a critical process in pulpitis as evident by the decayed tooth structure. Pulpitis is mainly caused by bacteria in dental caries that penetrate through the enamel and dentin to reach the pulp [40]. Among various oral bacteria,* P. intermedia* has gained attention due to a significant association between the bacterial load in carious dentin and periapical tooth disease [6]. In addition,* P. intermedia* LPS has unique chemical and immunobiological characteristics considerably different from those of the classical LPSs from* Escherichia coli* and* Salmonella* species [41–43]. Previous studies have shown that* P. intermedia* LPS induced several mediators of inflammation in human dental pulp cells [8, 9], murine macrophages [44], and bone cells [45] and demonstrated inflammatory responses in mice [46]. Our data show for the first time that LPS from* P. intermedia* would be able to upregulate the danger signals HMGB1 and RAGE in human dental pulp inflammation.

Dental pulp inflammation, especially in cases of excessive dentin damage or caries exposed pulp, does not resolve completely but becomes chronic with moderate inflammatory infiltrate, collagenous fibrosis, and may lead to pulp necrosis and dental abscess development [1, 6]. HMGB1 can be actively secreted into the extracellular space by activated pituicytes [47] or passively released from the nuclei of necrotic or damaged cells [48]. The active secretion of HMGB1 involves translocation from the nucleus to secretory lysosomes in the cytoplasm and then exocytosis [49]. In our study, we revealed a link between extracellular HMGB1 and LPS exposure. Specifically, that LPS induces HMGB1 translocation from the nucleus towards the cytoplasm and then secretion by OLC-1 cells in a dose- and time-dependent manner. Concentrations of LPS used for HMGB1 stimulation in this study were not cytotoxic to OLC-1 cells (data not shown). Our results on changes in HMGB1 in OLC-1 cells are consistent with those of previous studies in which HMGB1 is actively secreted from LPS-activated immune and nonimmune cells involved in chronic inflammatory diseases [50–52]. Furthermore, HMGB1 was solely localized in the nucleus of odontoblasts in healthy tissues, while a significant proportion of HMGB1 translocated to the cytoplasm in pulpitis tissue, which correlates with an increase in extracellular HMGB1 in LPS stimulated OLC-1 cells. In addition, our findings demonstrated only a distinct translocation of nuclear HMGB1 to the cytoplasm of odontoblasts but not pulp fibroblast-like cells in tissues from the pulpitis patients. Our results indicated that HMGB1 may be involved in the inflammatory response of teeth to bacteria in dental caries and that odontoblasts act as essential HMGB1-secretory cells in inflamed dental pulp tissues. In addition, in response to HMGB1 stimulation, human microvascular endothelial cells increase expression of RAGE and cell adhesion molecules, such as intercellular adhesion molecule (ICAM)-1 and vascular cell adhesion protein (VCAM)-1 and the secretion of proinflammatory cytokines including TNF*α* and IL-8 [19, 53]. Therefore, secreted HMGB1 from odontoblasts may contribute to the progression of human pulpitis pathogenesis.

Recent data have shown that LPS-mediated functions are conveyed via multiple receptors, such as RAGE [54], and several members of the Toll-like receptor (TLR) family including TLR2 and TLR4 [55]. In our study, upon challenge to* P. intermedia* LPS, RAGE mRNA and protein are upregulated in OLC-1 cells. Specifically, RAGE labeling appeared more intensely stained in odontoblasts of pulpitis versus healthy tissue. RAGE expression by odontoblasts appears comparable to that reported for alveolar epithelial cells [56], synovial fibroblasts [38], and in the pathogenesis of lung injury in mice [54, 57] in which RAGE is expressed at low levels in normal tissues and in the vasculature and becomes upregulated at other sites where its ligands accumulate [19]. Previous studies show that odontoblasts express receptors for LPS, TLR-2, and TLR-4 on the cell membrane [3, 58].* P. intermedia* induced IL-8 production is mediated by TLR-2 in activated monocytic cells [59] and TLR-2 mediated inflammatory responses to bacterial components in these cells [58, 60]. Our studies illustrate that HMGB1 release following* P. intermedia* LPS stimulation was suppressed by RAGE knockdown OLC-1, suggesting that overexpression of RAGE was essentially required for subsequent HMGB1 release, contributing to the progression of pulpitis. However, whether overlap exists of downstream events initiated by the cooperation of both TLR and RAGE in HMGB1 signaling in odontoblasts is unknown at present but is a focus of ongoing investigation.

RAGE is thought to be important in a variety of pathological conditions and is implicated in chronic inflammatory processes present in diabetes [61], rheumatoid arthritis [62], and Alzheimer's disease [63]. RAGE regulates inflammation though NF-*κ*B, AP-1, and Stat3 transcriptional regulation [64]. Biochemical and genetic studies have found that the IKK complex plays a critical role in the activation of NF-*κ*B. The IKK molecule consists mainly of two catalytic domains, IKK-*α* and IKK-*β*, and a noncatalytic chaperone protein IKK-*γ* [65]. The type of LPS used in our study induced stimulation of NF-*κ*B binding activity in previous studies [66]. Therefore, we elucidated the underlying mechanisms of the same LPS on HMGB1 expression. The pronounced increase of HMGB1 in the cytoplasmic fraction and supernatant of LPS stimulated cells was blocked by a potent inhibitor of IKK-*β* (TPCA-1). These results supported our hypothesis that* P. intermedia* LPS-mediated HMGB1 translocation involves NF-*κ*B activation in OLC-1. Our data are consistent with a previous study which demonstrated that IKK-*β* is essential for mediating the inflammatory response [67]. However, the present study does not exclude the possibility that other signaling pathways may be involved in activation of the RAGE/HMGB1 axis by LPS.

## 5. Conclusions

The particular cytokines released during chronic pulpal inflammation are of major importance with regard to the way in which the inflammation develops and is sustained. The continued expression of HMGB1 over time following inflammation can act, at least in part, as an important amplification signal for progressive pulpal destruction. The finding that HMGB1 acts as a proinflammatory mediator in pulpitis may provide new avenues for anti-inflammatory intervention, such as inhibiting the downregulation of dentin formation and production of proinflammatory molecules, thus suppressing dental pulp abscess formation. Therefore, understanding the mechanisms regulating the proinflammatory mediator HMGB1 and its receptor RAGE may lead to novel therapeutic approaches in pulpitis.

## References

[1] Farges JC, Alliot-Licht B, Baudouin C, Msika P, Bleicher F, Carrouel F (2013). Odontoblast control of dental pulp inflammation triggered by cariogenic bacteria. *Frontiers in Physiology*.

[2] Laurent P, Camps J, About I (2012). BiodentineTM induces TGF-*β*1 release from human pulp cells and early dental pulp mineralization. *International Endodontic Journal*.

[3] Veerayutthwilai O, Byers MR, Pham T-T, Darveau RP, Dale BA (2007). Differential regulation of immune responses by odontoblasts. *Oral Microbiology and Immunology*.

[4] Li P, Xue Y, Zhang W (2013). Sodium fluoride induces apoptosis in odontoblasts via a JNK-dependent mechanism. *Toxicology*.

[5] Haapasalo M, Ranta H, Ranta K, Shah H (1986). Black-pigmented Bacteroides spp. in human apical periodontitis. *Infection and Immunity*.

[6] Morrison DC, Ryan JL (1987). Endotoxins and disease mechanisms.. *Annual Review of Medicine*.

[7] Tokuda M, Sakuta T, Fushuku A, Torii M, Nagaoka S (2001). Regulation of interleukin-6 expression in human dental pulp cell cultures stimulated with *Prevotella intermedia* lipopolysaccharide. *Journal of Endodontics*.

[8] Nagaoka S, Tokuda M, Sakuta T (1996). Interleukin-8 gene expression by human dental pulp fibroblast in cultures stimulated with *Prevotella intermedia* lipopolysaccharide. *Journal of Endodontics*.

[9] Coil J, Tam E, Waterfield JD (2004). Proinflammatory cytokine profiles in pulp fibroblasts stimulated with lipopolysaccharide and methyl mercaptan. *Journal of Endodontics*.

[10] Stashenko P, Teles R, D'Souza R (1998). Periapical inflammatory responses and their modulation. *Critical Reviews in Oral Biology and Medicine*.

[11] Bletsa A, Berggreen E, Fristad I, Tenstad O, Wiig H (2006). Cytokine signalling in rat pulp interstitial fluid and transcapillary fluid exchange during lipopolysaccharide-induced acute inflammation. *Journal of Physiology*.

[12] Schmidt AM, Hofmann M, Taguchi A, Stern DM (2000). RAGE: a multiligand receptor contributing to the cellular response in diabetic vasculopathy and inflammation. *Seminars in Thrombosis and Hemostasis*.

[13] Schmidt AM, Stern DM (2001). Receptor for age (RAGE) is a gene within the major histocompatibility class III region: implications for host response mechanisms in homeostasis and chronic disease. *Frontiers in Bioscience*.

[14] Neeper M, Schmidt AM, Brett J (1992). Cloning and expression of a cell surface receptor for advanced glycosylation end products of proteins. *The Journal of Biological Chemistry*.

[15] Hori O, Brett J, Slattery T (1995). The receptor for advanced glycation end products (RAGE) is a cellular binding site for amphoterin. Mediation of neurite outgrowth and co-expression of RAGE and amphoterin in the developing nervous system. *The Journal of Biological Chemistry*.

[16] Hofmann MA, Drury S, Fu C (1999). RAGE mediates a novel proinflammatory axis: a central cell surface receptor for S100/calgranulin polypeptides. *Cell*.

[17] Bierhaus A, Humpert PM, Morcos M (2005). Understanding RAGE, the receptor for advanced glycation end products. *Journal of Molecular Medicine*.

[18] Schmidt AM, Yan SD, Yan SF, Stern DM (2001). The multiligand receptor RAGE as a progression factor amplifying immune and inflammatory responses. *The Journal of Clinical Investigation*.

[19] Fiuza C, Bustin M, Talwar S (2003). Inflammation-promoting activity of HMGB1 on human microvascular endothelial cells. *Blood*.

[20] Andersson U, Wang H, Palmblad K (2000). High mobility group 1 protein (HMG-1) stimulates proinflammatory cytokine synthesis in human monocytes. *Journal of Experimental Medicine*.

[21] Taniguchi N, Kawahara K, Yone K (2003). High mobility group box chromosomal protein 1 plays a role in the pathogenesis of rheumatoid arthritis as a novel cytokine. *Arthritis and Rheumatism*.

[22] Oyama Y, Hashiguchi T, Taniguchi N (2010). High-mobility group box-1 protein promotes granulomatous nephritis in adenine-induced nephropathy.. *Laboratory Investigation*.

[23] Kikuchi K, Miura N, Morimoto Y (2011). HMGB1 as a therapeutic target in spinal cord injury: a hypothesis for novel therapy development. *Experimental and Therapeutic Medicine*.

[24] Morimoto Y, Tancharoen S, Kikuchi K (2008). Tumor necrosis factor-*α* stimulates gingival epithelial cells to release high mobility-group box 1. *Journal of Periodontal Research*.

[25] Okuma Y, Wake H, Zhang J (2012). Anti-high mobility group box-1 antibody therapy for traumatic brain injury. *Annals of Neurology*.

[26] Kikuchi K, Tancharoen S, Ito T (2013). Potential of the angiotensin receptor blockers (ARBs) telmisartan, irbesartan, and candesartan for inhibiting the HMGB1/RAGE axis in prevention and acute treatment of stroke. *International Journal of Molecular Sciences*.

[27] Okuma Y, Liu K, Wake H (2012). Anti–high mobility group box-1 antibody therapy for traumatic brain injury. *Annals of Neurology*.

[28] Sawa H, Takeyama Y, Yasuda T, Shinzeki M, Nakajima T, Kuroda Y (2006). Blockade of high mobility group box-1 protein attenuates experimental severe acute pancreatitis. *World Journal of Gastroenterology*.

[29] Liu Z, Falo LD, You Z (2011). Knockdown of HMGB1 in tumor cells attenuates their ability to induce regulatory T cells and uncovers naturally acquired CD8 T cell-dependent antitumor immunity. *Journal of Immunology*.

[30] Tamura M, Tokuda M, Nagaoka S, Takada H (1992). Lipopolysaccharides of Bacteroides intermedius (*Prevotella intermedia*) and Bacteroides (*Porphyromonas*) gingivalis induce interleukin-8 gene expression in human gingival fibroblast cultures. *Infection and Immunity*.

[31] Hamada S, Koga T, Nishihara T, Fujiwara T, Okahashi N (1988). Characterization and immunobiologic activities of lipopolysaccharides from periodontal bacteria.. *Advances in Dental Research*.

[32] McLachlan JL, Sloan A, Cooper PR (2003). Gene expression analysis of genes of the dentine pulp complex in healthy and carious teeth. *Archives of Oral Biology*.

[33] McLachlan JL, Smith AJ, Bujalska IJ, Cooper PR (2005). Gene expression profiling of pulpal tissue reveals the molecular complexity of dental caries. *Biochimica et Biophysica Acta*.

[34] Arany S, Nakata A, Kameda T, Koyota S, Ueno Y, Sugiyama T (2006). Phenotype properties of a novel spontaneously immortalized odontoblast-lineage cell line. *Biochemical and Biophysical Research Communications*.

[35] Heid CA, Stevens J, Livak KJ, Williams PM (1996). Real time quantitative PCR. *Genome research*.

[36] Pichiule P, Schmidt A, Vannucci S (2007). Hypoxia-inducible factor-1 mediates neuronal expression of the receptor for advanced glycation end products following hypoxia/ischemia. *The Journal of Biological Chemistry*.

[37] Nawa Y, Kawahara K-I, Tancharoen S (2009). Nucleophosmin may act as an alarmin: implications for severe sepsis. *Journal of Leukocyte Biology*.

[38] He Z, Wang Z, Chen Y, Shen Q, Dai S (2013). HMGB1 acts in synergy with lipopolysaccharide in activating rheumatoid synovial fibroblasts via p38 MAPK and NF-*κ*B signaling pathways. *Mediators of Inflammation*.

[39] Podolin PL, Callahan JF, Bolognese BJ (2005). Attenuation of murine collagen-induced arthritis by a novel, potent, selective small molecule inhibitor of IkappaB kinase 2, TPCA-1 (2-[(aminocarbonyl)amino]-5-(4-fluorophenyl)-3-thiophenecarboxamide), occurs via reduction of proinflammatory cytokines and antigen-induced T cell proliferation. *Journal of Pharmacology and Experimental Therapeutics*.

[40] Karapanou V, Kempuraj D, Theoharides TC (2009). Oral neuroimmune network and mast cells. *European Journal of Inflammation*.

[41] Hamada S, Takada H, Ogawa T, Fujiwara T, Mihara J (1990). Lipopolysaccharides of oral anaerobes associated with chronic inflammation: chemical and immunomodulating properties.. *International Reviews of Immunology*.

[42] Kirikae T, Nitta T, Kirikae F (1999). Lipopolysaccharides (LPS) of oral black-pigmented bacteria induce tumor necrosis factor production by LPS-refractory C3H/HeJ macrophages in a way different from that of Salmonella LPS. *Infection and Immunity*.

[43] Hashimoto M, Asai Y, Tamai R, Jinno T, Umatani K, Ogawa T (2003). Chemical structure and immunobiological activity of lipid A from Prevotella intermedia ATCC 25611 lipopolysaccharide. *The FEBS Letters*.

[44] Choi EY, Choi J, Choi I, Kim S (2013). DHA sup presses Prevotella intermedia lipopolysaccharide -induced production of proinflammatory med iators in murine macrophages. *British Journal of Nutrition*.

[45] Pelt P, Zimmermann B, Ulbrich N, Bernimoulin J-P (2002). Effects of lipopolysaccharide extracted from *Prevotella intermedia* on bone formation and on the release of osteolytic mediators by fetal mouse osteoblasts in vitro. *Archives of Oral Biology*.

[46] Funayama H, Mayanagi H, Takada H, Endo Y (2001). Inflammatory reactions in extraoral tissues in mice after intragingival injection of lipopolysaccharide. *Journal of Infectious Diseases*.

[47] Wang H, Vishnubhakat JM, Bloom O (1999). Proinflammatory cytokines (tumor necrosis factor and interleukin 1) stimulate release of high mobility group protein-1 by pituicytes. *Surgery*.

[48] Scaffidi P, Misteli T, Bianchi ME (2002). Release of chromatin protein HMGB1 by necrotic cells triggers inflammation. *Nature*.

[49] Gardella S, Andrei C, Ferrera D (2002). The nuclear protein HMGB1 is secreted by monocytes via a non-classical, vesicle-mediated secretory pathway. *The EMBO Reports*.

[50] Chen Y, Gao R, Su Y, Umehara H, Dong L, Gong F (2013). The role of high mobility group box chromosomal protein 1 in rheumatoid arthritis. *Rheumatology (Oxford)*.

[51] Gardella S, Andrei C, Ferrera D (2002). The nuclear protein HMGB1 is secreted by monocytes via a non-classical, vesicle-mediated secretory pathway. *EMBO Reports*.

[52] Feghali K, Iwasaki K, Tanaka K (2009). Human gingival fibroblasts release high-mobility group box-1 protein through active and passive pathways. *Oral Microbiology and Immunology*.

[53] Chacur M, Milligan ED, Gazda LS (2001). A new model of sciatic inflammatory neuritis (SIN): induction of unilateral and bilateral mechanical allodynia following acute unilateral peri-sciatic immune activation in rats. *Pain*.

[54] Mangalmurti NS, Faust H, Lo R, Lee J, Worthen GS, Albelda SM (2013). LPS induced lung inflammation is mediated by the receptor for advanced glycation end products. *C22. New Insights into Acute Lung Injury*.

[55] Takeda K, Akira S (2005). Toll-like receptors in innate immunity. *International Immunology*.

[56] Yamakawa N, Uchida T, Matthay MA, Makita K (2011). Proteolytic release of the receptor for advanced glycation end products from in vitro and in situ alveolar epithelial cells. *The American Journal of Physiology—Lung Cellular and Molecular Physiology*.

[57] Zhang H, Tasaka S, Shiraishi Y (2008). Role of soluble receptor for advanced glycation end products on endotoxin-induced lung injury. *American Journal of Respiratory and Critical Care Medicine*.

[58] Durand SH, Roméas A, Carrouel F (2006). Lipoteichoic acid increases TLR and functional chemokine expression while reducing dentin formation in in vitro differentiated human odontoblasts. *Journal of Immunology*.

[59] Sugawara S, Yang S, Iki K (2001). Monocytic cell activation by nonendotoxic glycoprotein from *Prevotella intermedia* ATCC 25611 is mediated by toll-like receptor 2. *Infection and Immunity*.

[60] Botero TM, Shelburne CE, Holland GR, Hanks CT, Nör JE (2006). TLR_4_ mediates LPS-induced VEGF expression in odontoblasts. *Journal of Endodontics*.

[61] Ramasamy R, Yan SF, Schmidt AM (2011). Receptor for AGE (RAGE): signaling mechanisms in the pathogenesis of diabetes and its complications. *Annals of the New York Academy of Sciences*.

[62] Steenvoorden MMC, Toes REM, Ronday HK, Huizinga TWJ, DeGroot J (2007). RAGE activation induces invasiveness of RA fibroblast-like synoviocytes in vitro. *Clinical and Experimental Rheumatology*.

[63] Shi DY, Bierhaus A, Nawroth PP, Stern DM (2009). RAGE and Alzheimer’s disease: a progression factor for amyloid-*β*-induced cellular perturbation?. *Journal of Alzheimer's Disease*.

[64] Riehl A, Németh J, Angel P, Hess J (2009). The receptor RAGE: bridging inflammation and cancer. *Cell Communication and Signaling*.

[65] Ziegelbauer K, Gantner F, Lukacs NW (2005). A selective novel low-molecular-weight inhibitor of I*κ*B kinase-*β* (IKK-*β*) prevents pulmonary inflammation and shows broad anti-inflammatory activity. *British Journal of Pharmacology*.

[66] Tokuda M, Miyamoto R, Nagaoka S, Torii M (2004). Substance P enhances expression of lipopolysaccharide-induced inflammatory factors in dental pulp cells. *Journal of Endodontics*.

[67] Penzo M, Molteni R, Suda T (2010). Inhibitor of NK-*κ*B kinases *α* and *β* are both essential for high mobility group box 1-mediated chemotaxis. *The Journal of Immunology*.

